# 

**Published:** 1831

**Authors:** 


### ESSAYS AND CASES

| Art. | | Page. |
|---|---|---|
| I. | Essays on Medical Education, No. III. By the Senior Editor. - - - | 9 |
| II· | Hints on the Morality of Medicine. By Thomas D. Mitchell, Professor of Chemistry in Miami Uuiversity. - - - - - | 23 |
| III. | On the use of Mercury in Syphilis. By James M. Staughton, Professor of Surgery in Miami University. Read before the Cincinnati Medical Society. | 36 |
| IV. | Observations on Typhus Icterodes. By Robert J. Dodd, M. D., of the U. S. Navy. - - | 44 |
| V. | Extirpation of a Sarcomatous Testicle. By Dr. D. L. Saunders, of Perryville, Tenn. - - | 66 |
| VI. | The History of Lithotomy. By James M. Staughton, M. D., Professor of Surgery in Miami University. | 67 |

### REVIEWS AND BIBLIOGRAPHICAL NOTICES.

| | | |
|---|---|---|
| VII. | A Treatise on Hysteria. By George Tate, M. D. Member of the Royal College of Surgeons in London. - - - - - | 86 |
| VIII. | I. Journal of Health. Periodical. - Philadelphia, 1829. 2. The Catechism of Health; or Plain and Simple Rules for the Preservation of the Health and Vigor of the Constitution, from Infancy to Old Age. | 100 |
| IX. | Pharmacopœia of the United States, published by authority of the National Medical Convention held at Washington. - - - - | 119 |
| X. | A Dissertation on the Remote and Proximate Causes of Phthisis Pulmonalis. A Prize Essay. By A. Hammersly, M. D. - - - - | 121 |

### MISCELLANEOUS INTELLIGENCE.

### Analectic.

1. Experiments on Pulmonary Absorption and Exhalation. 121. 2. Observation on the influence of imperfect supplies of fresh air, long continued, on the general health. 124. 3. Phlebitis of the Saphena Veins, cured by Compression. 127. 4. Abdominal pressure in Obstetrics. 128. 5, Remarks on the operation for the Hare Lip. 6. Effects of Mercury. Taliacotian Operation. 129. 7. Acetate of Morphia in various diseases. 130. 8. Cause of Stammering. 132. 9. Itching of Delicate Surfaces. 135. 10. Effects of Posture on the Pulse, in Health and Disease. 136. 11. On the Compound Syrup and Extract Fluid of Sarsaparilla, 138. 12. Case of Putrefactive Disorganization of the Lungs. 140. 13. Gaseous Tumors of the Uterus. 141. 14. Small Pox communicated to the Fœtus in utero. 142. 15. Sulphureous Fumigations. 144. 16. New Pessary. 147. 17. Efficacy of Hydrocianic Acid in Curing Vomiting, not dependent upon Inflammation. 150. 18 Radical cure of Hernia. 151. 19. New Method for the cure of Hernia. 152. 20. Cases of Dislocation of the Thigh Bone, with remarks by the late Mr. Averill, Surgeon of Cheltenham. 153. 21. Remarks on the use of Injections of Warm Water, with a description of an Apparatus for their Administration. 156. 22. Lithotrity. 23. Section of the Sciatic Nerve. 160. 24. Wound of the Pericardium and Heart. 25. Vagitus Uterinus. 161. 26. New Symptoms of Pregnancy previous to the Fourth Mouth. 27. Medical Statistics: Report of the Coombe Lying-in Hospital, by Richard Reed Gregory. 162. 28. Davy and Wollaston compared as Philosophers. 165. 29. The Massachusetts Anatomy Bill. 166.

---

### NO. II.

### ESSAYS AND CASES.

| Art. | | Page. |
|---|---|---|
| I. | Essays on Medical Education, and the Medical Profession in the United States. By the Senior Editor. | 169 |
| II. | Experimental Researches on the Properties and Functions of the Nervous System, in Vertebrated Animals. By P. Flourens. Translated from the French. By the Junior Editor. - - - | 177 |

III. History of a case of Hydrophobia, not arising from an ascertained cause. By Daniel Drake, M. D. - 199

IV. Notes on a Fatal Case of Quiescent Gall Stones. By Daniel Drake, M. D. - - - 205

REVIEWS AND BIBLIOGRAPHICAL NOTICES.

V. Elements of Physics, or Natural Philosophy, General and Medical, explained independently of Technical Mathematics, and containing new disquisitions and practical suggestions. By Neil Arnott, M. D. of the Royal College of Physicians.—First American, from the third London edition; with additions, by Isaac Hays, A. M., M. D. &c. Philadelphia: Carey, Lea and Carey. pp. 532. - - - 211

VI. Select Medico-Chirurgical Transactions; or a collection of the Most Valuable Memoirs read to the Medico-Chirurgical Societies of London and Edinburgh, &c. &c. &c. Edited by Isaac Hays, M. D. 233

VII. A Treatise on Neuralgic Diseases, dependent upon Irritation of the Spinal Marrow and Ganglia of the Sympathetic Nerve. By Thomas Prigden, Teale 258

VIII. Lectures on the Theory and Practice of Surgery. By John Abernethy, F. R. S. - - - 274

MISCELLANEOUS INTELLIGENCE.

ANALECTIC.

1. Singular Trial for Infanticide. 280. 2. Can a woman, during the whole course of Utero Gestation, be ignorant that she is Pregnant? 284. 3. Medical Statistics of the Royal Maternity Charity, 286. 4, Are there certain Questions which a medical man in a court of Justice may refuse to answer? 289. 5. Mode of obtaining the active principle of the Elaterium. 291. 6. Process for economically preparing the Muriate of Morphia. 291. 7. Fracture of the edge of the Acetabulum. 292. 8. Fracture of the body of the Scapula. 295. 9. Dislocation of the Sacro-iliac Symphysis. 295. 10. Perforation of the Membrana Tympani. 297. 11. Wounds of the Throat. 298. 12. Trachetomy, 300. 13. Idiopathic Glossitis, 301. 14. On permanent involuntary contraction of the Muscles. 303. 15. Absorption of the Iris. 305. 16. New operation for the cure of Ptosis. 305. 17. On the energetic contractions of the Heart as a guide in the employment of Venesection. 306. 18. On the treatment of

Hooping Cough. 307. 19. Case of Constitutional Disease, arising from a Simple Local Irritation. 313. 20. Poisonous effect of the Blood of a Patient upon Leeches. 324. 21. Case of Spontaneous Lactation at an advanced age. 315. 22. Congenital absence of the Gall Bladder. 316. 23. Two newly discovered Muscles for compressing the Dorsal Vein of the Penis, in man and other animals. 316. 24. Taliacotian Operation for Restoration of the Under Lip. 317. 25. Pathology of Jaundice 319. 26. Living Skeleton. 319. 27. Poisonous Secretion from the body of a Horse. 320. 28. The use of Sulphate of Morphia in Opthalmia. 320. 20. Veratria and Colchicum Autumnale. 321. 30. The late Mr. Abernethy. 323. 31. Case in which brass wire was extracted from under the skin of a female, in whom it had induced Tetanic Symptoms. 328. 32. On a new practice in Acute and Chronic Rheumatism. 331. 33. Colchicum in Rheumatism. 335.

### NO. III.

### ESSAYS AND CASES.

| Art. | | Page. |
|---|---|---|
| I. | Observations upon the Nature and treatment of Parotitis Mercurialis. By George R. Morton, M. D., of Coshocton, Ohio - - - - | 337 |
| II. | History of a Sarcomatous Tumor, its extirpation, &c. Published by request of the Medical Board of the First Medical District of Indiana. By Washington W. Hitt, M. D., of Vincennes, Indiana - | 350 |
| III. | Cases of Spinal Irritation, producing and modifying the symptoms of disease. By Jas. C. Finley, M.D. | 354 |
| IV. | Elements of Chemistry, in the order of the Lectures given in Yale College, by Benjamin Silliman, Professor of Chemistry, Pharmacy, Mineralogy, and Geology, 2 vols., oct., p. 1262 - - - | 361 |
| V. | Researches, principally relative to the Morbid and Curative Powers of the Loss of blood. By Marshall Hall, M. D., F. R. S. &c. &c.—Philadelphia, Carey & Hart, 1830, pp. 173 - - - - - | 372 |
| VI. | Elements of Physics, or Natural Philosophy, General and Medical, explained independently of Technical Mathematics; and containing new disquisitions and practical suggestions. By Neil Arnott, M. D., of the | |

Royal College of Physicians. First American, from the third London edition, with additions. By Isaac Hays, A. M. M. D., &c.—Philadelphia; Carey, Lea & Carey, pp. 582 - - - 397

VII. Lectures on Physiology, Zoology, and the Natural History of Man, delivered at the Royal College of Surgeons. By W. Lawrence, F. R. S., Professor of Anatomy and Surgery to the College, &c. - 423

VIII. Practical Essays on Medical Education, and the Medical Profession in the United States. By Daniel Drake, M. D. - - - - 448

### MISCELLANEOUS INTELLIGENCE.

### I. Analectic.

1. Nature and Cure of the Indian Cholera. 449.—2. Several Cases of Spinal Irritation. 462.—3. Cases of Pulmonary Consumption. 467.—4. Intermittent Hemiplegia. 474.—5. Fatuity from an accumulation of Ascarides. 477.—6. Mode of arresting Hemorrhage from extraction of the Teeth. 478.—7. Lithotrity in Asia.—8. Case in which a large spoon was swallowed, and produced ulceration of the Duodenum and fatal Peritonitis. 479.—9. The Human Voice. 470.—10. Case of Poison by Rattlesnake. 480.—11. Medico-Legal Examination of two cases of sudden death from wounds. By Mr. Alexander Watson. 481.—12. Case of the successful treatment of Snake Bite by cupping glasses. (Transactions of the Medical and Physical Society of Calcutta; vol. 4. 487.—13. Culpable Abortion. 488.—14. Hæmatemesis cured by Emetics. 489.—15. Recto-Vesical Lithotomy. 491.—16. Mental Imbecility from injury of a nerve. 493.—17. Resuscitation. 494.—18. Tympanitic state of the Pericardium. 496.—19. Dropsy in Pregnancy. 497.

### II. Analytical.

1. On the origin of Voltaic Action.—2. Conducting power of Liquified Gasses. 497.—3. Generation of Steam by heated metal.—4. Spontaneous combustion of Pulverised Charcoal. 498.—5. To obtain Selerium from the Sulphuret.—6. Iodic Acid a test for the Vegeto-Alkalies.—7. Influence of occupation on the develepement of Phthisis Pulmonatis. 499—8. Twisting of the Arteries.—9. Lithotrity.—10. New Remedy. 501.—11. Acupuncturation of the Arteries.—12. Action of Oil of Turpentine, Opium, &c., on the Nervous System.—13. Lithotomy. 502.—14. Polypus.—15. Vanadium.—16. Magnesium. 503.—17. Phosphate of Quinine. 504.

### NO. IV.

### ESSAYS AND CASES.

ART. PAGE.

I. Essays on Medical Education and the Medical Profession in the United States. By Daniel Drake, M. D. 505

II. Observations of the Pathology and Treatment of Cholera Infantum. By John Wiltbank, M. D. of Phil'a. 530

III. Observations on the use and abuse of Emetics. By Daniel Drake, M. D. - - 543

IV. On the causes of Dyspepsia and other Diseases to which Students are liable. By Jas. C. Finley, M. D. 560

### REVIEWS AND BIBLIOGRAPHICAL NOTICES.

V. Lectures on Physiology, Zoology and the Natural History of Man. - - - 565

VI. Epidemic Cholera—Its Pathology and Treatment. An Eclectic Review.- - - 593—652

### MISCELLANEOUS INTELLIGENCE.

I. ANALECTIC.

1. An account of the origin, progress and present state of the Medical School of Paris. 617. 2. Effects of Opium Eating. 627. 3. Neuralgia successfully treated with the Cyanuret of Potassium. 630. 4. Excoriation of the Mamma. 5. Increased sensibility of the retina. 631. 6. On Poisoning with Acetic Acid 633. 7. Opium. 634. 8. Acetate of Morphia. 9. Piperine. 635. 10. Salacine. 11. Oil of Cantharadin. 12. Hermaphrodism. 636. 13. Extraordinary Abstinence. 14. On Inflammation of the Medullary Tissue of the long Bones. 637. 15. Tetanus from Inflammation of the Spinal Cord. 641. 16. Small Pox. 17. Pustules in the Intestines caused by the internal administration of Tartar Emetic. 18. Aneurism of the right Axillary Artery cured by Tying the Subclavian Artery. 642. 19. Is the meat of Diseased Animals unwholesome ? 643. 20. The Use of Trephine in Epilepsy. 646. 21. A case of Hereditary Hæmorrhagic Tendency. 947. 22. Analysis of the Sulphur Springs at Nashville. 23. A Class of Abortives. 648. 24. Tenacity of Vegetable Life. 25. On the Indentity of Small Pox and Cow Pox, and on a mode of inducing the Vaccine Pustule in the Cow at pleasure. 650. 26. Influence of Atmospheric Electricity on the Eyes. 651.

